# Gene Expression Profile Analysis in Epilepsy by Using the Partial Least Squares Method

**DOI:** 10.1155/2014/731091

**Published:** 2014-05-12

**Authors:** Dong Wang, Xixiao Song, Yan Wang, Xia Li, Shanshan Jia, Zhijing Wang

**Affiliations:** Department of Neurology, Xi'an Children's Hospital, Xi'an, Shaanxi 710003, China

## Abstract

*Purpose*. Epilepsy is a common chronic neurological disorder. We aim to investigate the underlying mechanism of epilepsy with partial least squares- (PLS-) based gene expression analysis, which is more sensitive than routine variance/regression analysis. *Methods*. Two microarray data sets were downloaded from the Gene Expression Omnibus (GEO) database. PLS analysis was used to identify differentially expressed genes. Gene ontology and network analysis were also implemented. *Results*. A total of 752 genes were identified to be differentially expressed, including 575 depressed and 177 overexpressed genes in patients. For GO enrichment analysis, except for processes related to the nervous system, we also identified overrepresentation of dysregulated genes in angiogenesis. Network analysis revealed two hub genes, *CUL3* and *EP300*, which may serve as potential targets in further therapeutic studies. *Conclusion*. Our results here may provide new understanding for the underlying mechanisms of epilepsy pathogenesis and will offer potential targets for producing new treatments.

## 1. Introduction

Epilepsy is a common chronic neurological disorder, which has devastating effects on patients and their families. There are about 50 million epilepsy patients worldwide and the occurrence in developing countries is more than twice that in developed countries [1]. Currently there are more than 20 antiepileptic drugs available for epilepsy patients [2]; however, multidirectional interactions between seizures and the medications are still challenging for treating patients [3]. Exploring the biological alterations of patients may provide insights into the pathology and new targets for treatments.

Large-scale microarray expression strategy has provided greater ease for investigating the underlying mechanisms of epilepsy. Several gene expression profiling studies have been carried out earlier and most of them used the routine variance/regression analysis [4–6]. However, this procedure cannot remove unaccounted array specific factors, such as certain demographic profiles. Compared with the routine analysis, previous studies [7, 8] proposed that partial least squares- (PLS-) based analysis is more robust in proceeding gene expression profile data with higher sensitivity. Therefore, using PLS analysis may provide new understanding of the pathogenesis of epilepsy.

In the current study, to identify truly differentially expressed genes between epilepsy patients and normal controls, we carried out a PLS analysis with two combined data sets from the Gene Expression Omnibus (GEO) database. Gene ontology (GO) enrichment analysis was also carried out for the selected genes to capture the biological relevant signatures. A network constructed by proteins encoded by dysregulated genes was used to identify key molecules among the differentially expressed genes. Our results here may provide new understanding on the pathogenesis of epilepsy.

## 2. Materials and Methods

### 2.1. Microarray Data

Two data sets (GSE4290 and GSE50161) from the GEO (http://www.ncbi.nlm.nih.gov/geo/) database, which include 23 epilepsy patients and 13 healthy controls, were used in this study.

The two data sets were both based on the GPL570platform Affymetrix Human Genome U133 Plus 2.0 Array. Detailed information of the samples is listed in Table 1.

### 2.2. Detection of Differentially Expressed Genes

Normalization of raw intensity values was carried out with robust multiarray analysis (RMA) [9]. The resulting log2-transformed expression values of all probes were used for further PLS analysis [10, 11], which is a dimension reduction method for modeling without imposing strong assumptions, to estimate the effects for each probe in epilepsy patients. Briefly, NIPALS algorithm [12] was firstly used to obtain PLS latent variables derived from the expression profile; variable importance on the projection (VIP) [13] was then calculated to estimate the effect of the expressed probes on the disease status of the patients. Finally, the empirical distribution of PLS-based VIP was obtained with a permutation procedure (*N* = 10000 times) and false discovered rate (FDR) of each probe was calculated based on the empirical distribution. Probes with FDR value less than 0.05 were selected as differentially expressed genes in this study.

### 2.3. Enrichment Analysis

Identified differentially expressed probes were annotated by using the simple omnibus format in text (SOFT) format files. All genes were then mapped to the Gene Ontology database [14], which provides a controlled vocabulary of terms for describing gene product characteristics. Hyper geometric distribution test was carried out to identify GO items enriched with differentially expressed genes.

### 2.4. Network Analysis

Most proteins function through interactions with other proteins. Proteins with more interactions with other proteins are supposed to play more important roles in biological processes. To identify key molecules among the differentially expressed genes, we constructed an interaction network with the proteins encoded by selected genes by using the software Cytoscape (V 2.8.3, http://www.cytoscape.org/) [15]. Interaction information of the proteins was obtained from the NCBI database (http://ftp.ncbi.nlm.nih.gov/gene/GeneRIF/). The number of links (interactions) for each protein was defined as its degree. Proteins with degrees more than 10 were selected as hub molecules in this study.

## 3. Results

**Figure 1:**
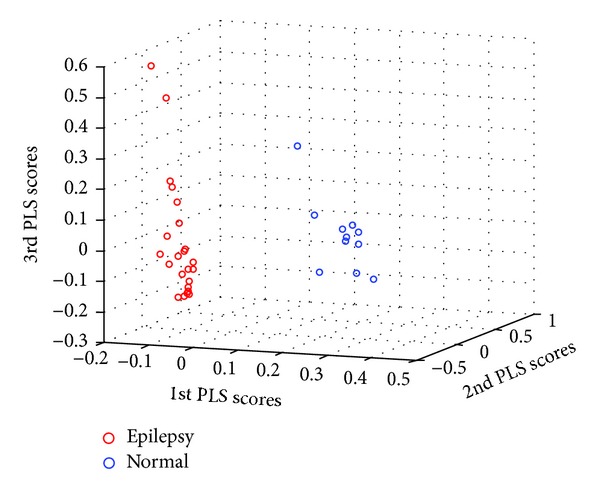
Sample classification by using the three selected partial least squares (PLS) latent variables.

After quality control, two samples (GSM1214938 and GSM1214939) were excluded from subsequent analysis due to aberrant RNA degradation. Thus, 23 epilepsy patients and 11 healthy controls were used in PLS analysis. Sample classification according to the three selected latent variables is illustrated in Figure 1. After FDR control, a total of 752 genes were identified to be differentially expressed, including 575 depressed and 177 overexpressed genes in patients. The top ten GO items enriched with differentially expressed genes are listed in Table 2. Most of them (60%) are related to the nervous system, including nervous system development (GO:0007399), central nervous system myelin maintenance (GO:0007399), neuroligin clustering (GO:0007399), synapse assembly (GO:0007416), spinal cord motor neuron differentiation (GO:0021522), and glial cell development (GO:0021782).

**Figure 2:**
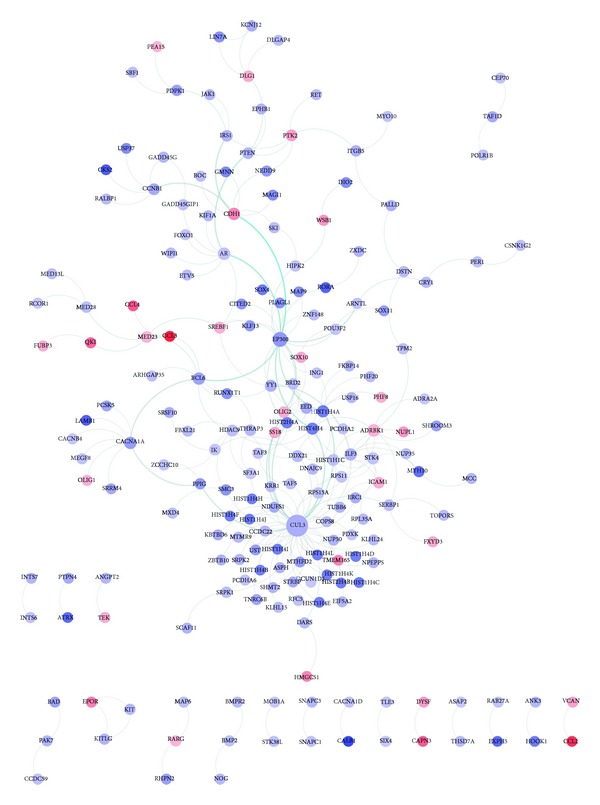
Interaction network constructed with proteins encoded by selected genes. Proteins with more links are shown in bigger size. Proteins shown in red are encoded by overexpressed genes in patients while those in blue are encoded by depressed genes in patients.

Figure 2 represents the interaction network of proteins encoded by selected genes. Two proteins, CUL3 and EP300, were identified to be hub molecules, with degrees of 56 and 21, respectively.

## 4. Discussion

Pathophysiology of epilepsy is highly complex. Gene expression profiling is useful in investigating the underlying mechanism of epilepsy. For the data analysis, creating a suitable model to handle small sample sizes and large number of genes [7] remains challenging. Previous studies [7, 8] have demonstrated better performance of the PLS-based method than common variance/regression analysis, which cannot remove hidden biological effects. Here we used PLS analysis to identify differentially expressed genes between epilepsy patients and healthy controls.

As shown in Figure 1, the selected three latent variables performed well in classification of the samples. GO enrichment analysis of the selected genes revealed the overrepresentation of differentially expressed genes in the nervous system. This is consistent with previous studies. For example, glial cells have been reported to play prominent roles in seizure precipitation and recurrence [16], and the glial cell development was identified to be enriched with dysregulated genes in our study (*P* = 4.18 × 10^−3^). In addition, angiogenesis (GO:0001525) was also found to be overrepresented with dysregulated genes. Dysregulation of angiogenesis may be related to the dysfunction of blood-brain barrier, contributing to epileptogenesis [17]. Signs of angiogenesis were also reported to be corresponding with seizure-induced neuronal death in animal models of familial epilepsy [18]. Our results here further confirmed the involvement of angiogenesis process in the pathogenesis of epilepsy.

According to the network analysis, CUL3 was identified to be a hub molecule with the highest degree (Figure 2). CUL3 is a core component and scaffold protein of an E3 ubiquitin ligase complex. Previous expression studies have not reported the differential expression of CUL3 in epilepsy patients. However, E3 ubiquitin ligase may affect the synaptic functions in the central nervous system and the stability of kainate receptors, which form a class of glutamate receptors implicated in epilepsy [19, 20]. Our results suggested that CUL3 may serve as a potential target in therapeutic studies.

*EP300* is another hub gene with the degree of 21. No report of this gene and epilepsy has been proposed before. However, protein encoded by this gene is a transcriptional coactivator, which stimulates CREB-dependent gene expression. Seizure disorder was reported to be more frequent in Rubinstein-Taybi syndrome patients with CREBBP mutations [21]. In addition, the promoted signaling mechanism of EP300 is important in the neuronal survival process [22] and this gene was related to other neuronal disorders, such as familial Alzheimer's disease [22]. Thus, the correlation of this gene and epilepsy pathogenesis may involve the activity of CREB-dependent proteins and further investigation is warranted.

In summary, using two data sets from the GEO database, we carried out PLS-based gene expression analysis to investigate the underlying pathology of epilepsy. Except for processes related to the nervous system, we also identified overrepresentation of dysregulated genes in angiogenesis. Network analysis revealed two hub genes,* CUL3* and* EP300*, which may serve as potential targets in further therapeutic studies. Our results here may provide new understanding for the underlying mechanisms of epilepsy pathogenesis and will offer potential targets for producing new treatments.

## Figures and Tables

**Table 1:** Characteristics of the expression profile used in this study.

Accession ID	Description
GSM97800	Brain tissue from epilepsy patient
GSM97803	Brain tissue from epilepsy patient
GSM97804	Brain tissue from epilepsy patient
GSM97805	Brain tissue from epilepsy patient
GSM97807	Brain tissue from epilepsy patient
GSM97809	Brain tissue from epilepsy patient
GSM97811	Brain tissue from epilepsy patient
GSM97812	Brain tissue from epilepsy patient
GSM97816	Brain tissue from epilepsy patient
GSM97817	Brain tissue from epilepsy patient
GSM97820	Brain tissue from epilepsy patient
GSM97825	Brain tissue from epilepsy patient
GSM97827	Brain tissue from epilepsy patient
GSM97828	Brain tissue from epilepsy patient
GSM97833	Brain tissue from epilepsy patient
GSM97834	Brain tissue from epilepsy patient
GSM97840	Brain tissue from epilepsy patient
GSM97846	Brain tissue from epilepsy patient
GSM97848	Brain tissue from epilepsy patient
GSM97849	Brain tissue from epilepsy patient
GSM97850	Brain tissue from epilepsy patient
GSM97853	Brain tissue from epilepsy patient
GSM97855	Brain tissue from epilepsy patient
GSM1214936	Brain tissue from normal control
GSM1214937	Brain tissue from normal control
GSM1214938	Brain tissue from normal control
GSM1214939	Brain tissue from normal control
GSM1214940	Brain tissue from normal control
GSM1214941	Brain tissue from normal control
GSM1214942	Brain tissue from normal control
GSM1214943	Brain tissue from normal control
GSM1214944	Brain tissue from normal control
GSM1214945	Brain tissue from normal control
GSM1214946	Brain tissue from normal control
GSM1214947	Brain tissue from normal control
GSM1214948	Brain tissue from normal control

**Table 2:** The top 10 GO items enriched with differentially expressed genes.

GO_id	GO_description	GO_class	*P* value
GO:0007155	Cell adhesion	Process	3.74*E* − 05
GO:0072659	Protein localization to plasma membrane	Process	4.75*E* − 05
GO:0001525	Angiogenesis	Process	2.64*E* − 04
GO:0007399	Nervous system development	Process	9.26*E* − 04
GO:0032286	Central nervous system myelin maintenance	Process	1.43*E* − 03
GO:0097118	Neuroligin clustering	Process	1.43*E* − 03
GO:0007416	Synapse assembly	Process	1.59*E* − 03
GO:0021522	Spinal cord motor neuron differentiation	Process	2.57*E* − 03
GO:0005509	Calcium ion binding	Function	4.01*E* − 03
GO:0021782	Glial cell development	Process	4.18*E* − 03
